# Mental health in society’s margins: poor *n*-3 PUFA intake and psychological well-being of homeless youth

**DOI:** 10.1017/S000711452300212X

**Published:** 2024-02-28

**Authors:** Sarah Beth Dunn, Tonya S. Orchard, Rebecca Andridge, Susan M. Rymut, Natasha Slesnick, Irene E. Hatsu

**Affiliations:** 1 Human Nutrition Program, Department of Human Sciences, The Ohio State University, Columbus, OH 43210, USA; 2 Division of Biostatistics, College of Public Health, The Ohio State University, Columbus, OH, USA; 3 Human Development and Family Science Program, Department of Human Sciences, The Ohio State University, Columbus, OH, USA; 4 OSU Extension, The Ohio State University, Columbus, OH, USA

**Keywords:** *n*-3 PUFA intake, *n*-3 PUFA status, Psychological well-being, Homeless youth, Mental health

## Abstract

Dietary intake of long-chain *n*-3 PUFA (*n*-3 PUFA), particularly EPA and DHA, has been associated with psychological well-being, but little is known about the *n*-3 PUFA intake of homeless youth. The current study determined the association between depression and anxiety symptoms and *n*-3 PUFA intake and erythrocytes status in homeless youth. Totally, 114 homeless youth aged 18–24 years were recruited from a drop-in centre. *n*-3 PUFA dietary intake was assessed using an FFQ, and erythrocytes status was determined by gas chromatography (GC). Linear regression models were used to determine the relationship between psychological well-being and *n*-3 PUFA intake and status. The mean intakes of EPA and DHA for all participants (0·06 ± 0·13 g/d and 0·11 ± 0·24 g/d) were well below recommended levels, and mean erythrocytes EPA + DHA (*n*-3 index) in the cohort (2·42 %) was lower than reported for healthy, housed adolescents and those with clinical depression. There was no association of *n*-3 PUFA intake and erythrocytes status with either depression or anxiety. However, the relationships of depression with dietary EPA (*P* = 0·017) and DHA (*P* = 0·008), as well as erythrocytes DHA (*P* = 0·007) and *n* 3-index (*P* = 0·009), were significantly moderated by sex even after adjusting for confounders. Specifically, among females, as the intake and status of these *n*-3 PUFA decreased, depression increased. Our findings show poor dietary intake and low erythrocytes status of *n*-3 PUFA among homeless youth, which is associated with depressive symptoms among females.

On any given night in the USA, it is estimated that approximately 34 000 youth are homeless and unaccompanied^(1)^. These individuals are not part of a homeless family, and many leave home for the first time around an age of 14·8 years^(2)^. Unfortunately, once on the street, these youth are often subjected to victimisation, abuse and crime^(3–5)^. Unsurprisingly then, whether a consequence of or contributor to homelessness, homeless youth are also more likely to experience poor mental health compared with housed peers^(6)^: an estimated 23–74 % of unaccompanied homeless youth have at least one mental disorder, the most common being major depressive disorders, anxiety, conduct disorder, post-traumatic stress disorder and bipolar disorders^(7,8)^.

On the street, homeless youth are challenged to find adequate resources^(9)^. Competing demands such as generating income, obtaining transportation and finding housing leave little time for food acquisition, compromising the youths’ ability to meet their nutritional needs^(9)^. As a result, many homeless youth do not consume adequate amounts of food and/or nutrients^(10–12)^. Previous diet studies in homeless youth indicate a low consumption of fruit, vegetables and whole grains, resulting in inadequate intakes of several vitamins and minerals (vitamins A, C, D_3_ and E as well as Ca and Mg)^(11,13,14)^, which can be harmful during periods of substantial growth and development like adolescence. Furthermore, when food is available, it is typically inexpensive, energy-dense packaged or fast food^(9,15,16)^. Difficulties meeting nutritional needs may also be compounded in homeless youth with poor mental health^(17,18)^, as studies have found these individuals generally consume more calories, carbohydrates, added sugars and fats than their peers without mental health disorders^(19)^. Limited evidence exists considering the intake of essential fatty acids among homeless youth, but it may be hypothesised that intakes of *n*-3 PUFA, specifically the long-chain *n*-3 PUFA, EPA and DHA, are low due to the large amount of processed and packaged foods consumed by this population.

Mental health has been associated with PUFA, especially DHA, because of the role fatty acids play in the brain^(20)^. Grey matter and synapses are highly saturated in DHA and other *n*-3 PUFA^(20)^, and approximately 4·6 mg of DHA are used by the brain each day^(21)^. Since body concentrations of PUFA are generally conserved in adulthood^(22)^, this amount plus DHA needed by other organ systems must be replenished by food or by the conversion of *α*-linolenic acid (ALA) or EPA to DHA. However, conversion of ALA to EPA and DHA is small in humans (estimated at 10 % in males and 14 % in females) and is dependent on the background diet of the individual^(23–25)^. Seafoods (e.g. fatty fish and shellfish) are the primary source of the long-chain *n*-3 FA EPA and DHA in the human diet^(25)^, but these foods are generally expensive^(26)^, require preparation and mostly unavailable in places where homeless youth access food. Therefore, extensive barriers exist for adequate *n*-3 PUFA consumption in homeless youth. Considering the impact of diet on mental health, several studies, but not all, have shown that a higher consumption of fish rich in *n*-3 PUFA was associated with fewer depressive symptoms^(27–29)^. Additionally, postmortem studies have found that individuals with severe depression have lower DHA concentrations in their orbitofrontal cortex compared with controls without mood disorders^(30)^. Although there is evidence to support a correlation between PUFA status and mental health in the general population, it is not known if this association is present among homeless youth^(31)^.

The objective of this project was to quantify long-chain PUFA dietary intake and erythrocytes status among homeless youth, as well as determine whether *n*-3 PUFA relates to depression and anxiety. Our central hypothesis is that depression and anxiety will be inversely associated with intake and biological levels of *n*-3 PUFA (i.e. EPA, docosapentaenoic acid and DHA). Though homeless youth are subject to poor diet quality and stressful environmental factors, no known research to date has explored the association of *n*-3 PUFA and mental health in this population.

## Methods

### Study population and eligibility

This cross-sectional, observational study utilised survey data and biological samples from 114 homeless and unaccompanied youth recruited from a drop-in center in a large midwestern city in the USA who have been described previously in a study of other nutritional vulnerabilities^(13)^. This additional analysis is the first to analyse biological samples collected during this study. Homeless individuals, as defined in the McKinney-Vento Homeless Assistance Act (2002)^(32)^, included ‘those who lack a fixed, regular and adequate nighttime residence’. Eligibility for study inclusion were: (1) meeting the criteria for homelessness, as defined by the McKinney-Vento Act (2002); (2) being aged 18 to 24 years; and (3) understanding and signing an informed consent form. Exclusion criteria for the study included self-reported pregnancy due to the physiological and dietary changes that occur during this time.

Participants were recruited by flyer and word-of-mouth. After providing informed consent, potential participants completed a screening questionnaire, and those who met the inclusion criteria were enrolled in the study. Participants completed questionnaires on sociodemographic factors, homeless experience, dietary intake and mental health status, as well as provided a venous blood sample for fatty acid quantification. Participants were compensated $25 following the completion of all questionnaires, assessments and biological sampling.

### Ethical approval

The current study was conducted according to the guidelines laid down in the Declaration of Helsinki and all procedures involving human participants were approved by the Ohio State Behavioral and Social Sciences IRB (Protocol #2015H0265). Written informed consent was obtained from all participants.

### Measures

#### Sociodemographic measures

Self-administered paper questionnaires were used to collect demographic data such as age, sex, race, ethnicity, education and employment status. Homeless characteristics, such as the age of first homelessness and length of current homelessness, were also recorded.

#### Nutrient intake

The Block Food Frequency Questionnaire for Adults^(33)^, which is based on approximately 127 food items, was used to quantify the nutrient intake of all participants. A trained interviewer introduced the self-administered questionnaire, answered questions and read the questionnaire to the participant if literacy was limited.

#### Mental and overall health assessments

The Beck Depression Inventory Second Edition (BDI-II)^(34)^ and the Beck Anxiety Inventory (BAI)^(35)^ were completed by each participant to assess depression and anxiety symptoms, respectively. These are both validated twenty-one question, self-reported inventories for individuals at least 13 years of age to characterise symptom severity, not to diagnose^(34,35)^. Responses for both inventories were rated and totaled, with possible final scores ranging from 0–63. Higher scores on these assessments indicate more severe symptoms of depression and anxiety, respectively^(34)^. Specifically, the predetermined cut-off used were as follows: depression (0–13: minimal, 14–19: mild, 20–28: moderate, 29–63: severe) and anxiety (0–7: minimal, 8–15: mild, 16–25: moderate, 26–63: severe)^(34,35)^.

#### Biochemical assessment and lipid extraction

Fatty acid profiles were determined using gas chromatography after lipid extraction and methylation of erythrocytes. Erythrocytes fatty acids are not sensitive to short term dietary changes, have little biological variability compared with plasma fatty acids and represent PUFA status over several months^(36)^, making them an appropriate biomarker of long-chain PUFA intake for this study. Additionally, the sum of erythrocytes EPA + DHA, known as the *n*-3 Index, is a potential risk factor for chronic disease, including bipolar disorder in adolescents^(37,38)^. A non-fasted venous blood sample (40 ml) was taken from each participant and filled into vials containing anticoagulants. The sample was centrifuged within 1-h post-collection, and erythrocytes were aliquoted into microcentrifuge tubes. Specimens were stored at −80^o^C until analysis.

Using standard procedures previously described by Harris *et al.*^(39)^, fatty acids were extracted and methylated from erythrocytes samples. At the time of analysis, aliquots of erythrocytes were thawed on ice and 50 µl transferred to a glass tube containing 500 µl 14 % boron trifluoride (BF_3_) in methanol with butylated hydroxytoluene (Sigma Aldrich, St. Louis, MO). The sample was vortexed for 30 s followed by the addition of 500 µl of hexane and heating at 100^o^C in a bead bath for 10 min. After cooling to room temperature, 500 µl deionised water was added, and the samples were vortexed. They were then centrifuged for 3 min at 3000 g and 25^o^C. The top hexane phase containing the fatty acid methyl esters was transferred into a glass vial using a glass pipette and stored at −20^o^C until gas chromatography. The samples were analysed on a gas chromatograph (Shimadzu, Columbia, MD) using a 30-m capillary column (Omegawax™ 320, Supelco-Sigma). Retention times were compared with authentic standards for fatty acid methyl esters (Supelco-Sigma, St. Louis, MO and Matreya, Inc., Pleasant Gap, PA), and fatty acids were reported as a percentage of total fatty acids identified in the sample.

#### Statistical analysis

Descriptive statistics (reported as means and standard deviation or as proportions) were used to summarise participants’ psychological well-being (from the BDI-II and BAI questionnaires) as well as their long-chain PUFA intake and erythrocytes PUFA status. Independent sample *t*-tests were used to determine differences in means by sex. The sex-based subgroup analysis was predicated on previous research that showed sex-based differential nutrient intake and diet quality among homeless youth^(13)^, as well as the sex differences in the regulation and metabolism of *n*-3 fatty acids^(40)^. Depression and anxiety scores from the BDI-II and BAI were initially categorised into minimal, mild, moderate or severe for descriptive purposes, with the categories further collapsed into three groups (minimal, mild and moderate/severe) for modeling purposes. Dietary fatty acid intake was energy adjusted for use in linear regression models. Linear regression models were used to determine associations of dietary and erythrocytes *n*-3 PUFA status (dependent variables) with psychological well-being (independent variables: 3-level BDI-II and 3-level BAI in separate models), adjusting for age, sex, age first homeless and number of months without shelter, based on prior research^(13)^. To test for moderation of psychological well-being effects by sex, interactions between BDI-II/BAI and sex were added to these models. Statistical analyses were conducted using R (https://www.R-project.org). Because sample size was determined by the feasibility of recruitment, it was determined that a sample size of 114 would detect a large effect size (*f*2 = 0·337) at 0·90 power and an *α* of 0·05^(41)^.

## Results

### Participant characteristics

The average age of study participants was 21·4 ± 1·8 years, and 70 % of participants were male. About half of the participants (49 %) identified as Black/African American, 28 % identified as White and 17 % identified as ‘other’. The average BMI (Body Mass Index) for the sample was 27·8 ± 8·3 kg/m^2^, though females had a significantly higher BMI (32·5 ± 9·9 kg/m^2^) compared with males (25·8 ± 6·7 kg/m^2^; *P* = 0·007). Approximately two-thirds of participants (62 %) had a high school diploma or GED, 27 % had no diploma and only 4 % had an associate, technical or vocational degree (Table 1). Most youth were first homeless after the age of 18 (75 %), with the average age of first homelessness being 18·2 ± 3·1 years. However, 18 % were homeless between the ages of 15 and 17 and a further 6 % at 14 years of age or younger. About 42 % of study participants reported having been homeless for over 4 months. Reasons for current homelessness included financial instability and unemployment, arguments with family and friends, being thrown out and personal choice.

**Table 1.** Participant characteristics

Characteristics	Total (*n* 114)		Male (*n* 80)		Female (*n* 34)	
	Mean	sd	Mean	sd	Mean	sd
Age	21·4	1·8	21·4	1·7	21·4	1·9
BMI* (kg/m^2^)	27·8	8·3	25·8	6·7	32·5	9·9
	*n*	%	*n*	%	*n*	%
Age First homeless						
14 years or younger	7	6·1	5	6·3	2	5·9
15–17 years	21	18·4	16	20·0	5	14·7
18 years or older	86	75·4	59	73·8	27	79·4
Race						
African national	1	0·9	1	1·3	0	0
Asian American	3	2·6	2	2·5	1	2·9
Black/African American	56	49·1	38	47·5	18	52·9
Native American or Alaskan Native	2	1·8	1	1·3	1	2·9
White	32	28·1	22	27·5	10	59·4
Other	19	16·7	15	18·8	4	11·8
No Response	1	0·9	1	1·3	0	0
Education						
Still in high school	2	1·8	1	1·3	1	2·9
High school diploma	61	53·5	40	50·0	21	61·8
GED†	10	8·8	7	8·8	3	8·8
Associates degree	2	1·8	1	1·3	1	2·9
Some college	6	5·3	3	3·8	3	8·8
Vocational/Technical degree	2	1·8	2	2·5	0	0
No degree	31	27·2	26	32·5	5	14·7
Length of current homelessness						
> 4 months	48	42·1	36	45·0	12	35·3
≤ 4 months	66	57·9	44	55·0	22	64·7

* BMI.

† General educational development test.

### Dietary fatty acid intake

Table 2 shows the dietary fatty acid profile obtained from self-reported FFQ data (not energy-adjusted). The typical daily intake of select n-3 PUFA were 24·65 ± 26·77 g linoleic acid (LA)/d, 0·20 ± 0·23 g arachidonic acid (ARA)/d, 0·06 ± 0·13 g EPA/d and 0·11 ± 0·24 g DHA/d. Males and females had similar PUFA intakes except for ARA; males reported consuming significantly more ARA than females (0·24 ± 0·24 *v*. 0·13 ± 0·18 g/d; *P* = 0·043).

**Table 2.** Dietary fatty acid intake from FFQ

Fatty acid (g/d)	Total (*n* 100)		Male (*n* 70)		Female (*n* 30)		*P* value†
	Mean	sd	Mean	sd	Mean	sd	
Total fat	136·52	138·92	155·13	148·49	93·12	103·12	0·040*
SFA	45·37	43·78	51·83	46·58	30·31	32·34	0·023*
MUFA	51·25	53·52	58·34	57·14	34·71	40·08	0·042*
PUFA	27·85	30·25	31·27	32·64	19·89	22·28	0·085
LA (C18:2*n*-6)	24·65	26·77	27·63	28·88	17·69	19·77	0·089
ARA (C20:4*n*-6)	0·20	0·23	0·24	0·24	0·13	0·18	0·043*
ALA (C18:3*n*-3)	2·42	2·76	2·75	3·04	1·65	1·76	0·068
EPA (C20:5*n*-3)	0·06	0·13	0·06	0·13	0·06	0·14	0·996
DPA *n*-3 (C22:5*n*-3)	0·03	0·05	0·03	0·05	0·03	0·05	0·524
DHA (C22:6*n*-3)	0·11	0·24	0·11	0·24	0·11	0·25	0·902

ARA, arachidonic acid; LA, linoleic acid; ALA, *α*-linolenic acid; DPA, docosapentaenoic acid.

* *P* < 0·05.

† Two-sample *t*-test of fatty acid intake.

Fourteen participants had no dietary intake data hence *n* 100.

### Erythrocytes fatty acid profile

The mean erythrocytes EPA + DHA composition (the *n*-3 index) was 2·42 % (EPA: 0·28 ± 0·17 % and DHA: 2·14 ± 0·57 %). Mean compositions for ALA and docosapentaenoic acid *n*-3 were 0·54 ± 0·63 % and 1·77 ± 0·33 %, respectively. There were no significant differences in fatty acid percentages by sex except DHA, with females having a higher mean concentration of DHA than males (females: 2·37 ± 0·70 %; males: 2·04 ± 0·48 %; *P* = 0·005) (Table 3).

**Table 3.** Percent of total fatty acids in erythrocytes

Fatty acid	Total (*n* 114)		Male (*n* 80)		Female (*n* 34)		*P* value†
	Mean	SD	Mean	SD	Mean	SD	
LA (C18:2*n*-6)	15·68	2·44	15·61	2·55	15·83	2·21	0·670
GLA (C18:3*n*-6)	0·11	0·11	0·10	0·11	0·13	0·11	0·167
ALA (C18:3*n*-3)	0·54	0·63	0·55	0·68	0·53	0·49	0·896
EDA (C20:2*n*-6)	0·29	0·14	0·27	0·15	0·32	0·11	0·123
DGLA (C20:3*n*-6)	1·62	0·36	1·63	0·37	1·60	0·33	0·778
ARA (C20:4*n*-6)	15·10	1·88	15·0	1·9	15·3	1·7	0·461
EPA (C20:5*n*-3)	0·28	0·17	0·26	0·18	0·31	0·16	0·182
DTA (C22:4*n*-6)	3·62	0·66	3·63	0·65	3·63	0·65	0·851
DPA *n*-6 C22:5*n*-6	0·51	0·24	0·51	0·25	0·52	0·20	0·894
DPA *n*-3 (C22:5*n*-3)	1·77	0·33	1·75	0·34	1·81	0·31	0·405
DHA (C22:6*n*-3)	2·14	0·57	2·04	0·48	2·37	0·70	0·005**

LA, linoleic acid; GLA, *γ*-linolenic acid; ALA, *α*-linolenic acid; EDA, eicosadienoic acid; DGLA, dihomo-*γ*-linolenic acid; ARA, arachidonic acid; DTA, docosatetraenoic acid; DPA, docosapentaenoic acid.

† Two-sample *t*-test of fatty acid erythrocytes status.

** *P* < 0·01.

Mean CV from duplicate or triplicate samples was 3·4 % for LA, 6·6 % for ALA, 2·7 % for EPA and 2·9 % for DHA. ALA was below the limit of detection (LOD) for fourteen participants, and EPA was below the LOD for twenty-seven participants. Not all participants that had ALA below LOD also had EPA below LOD.

### Depression and PUFA

**Fig. 1.**
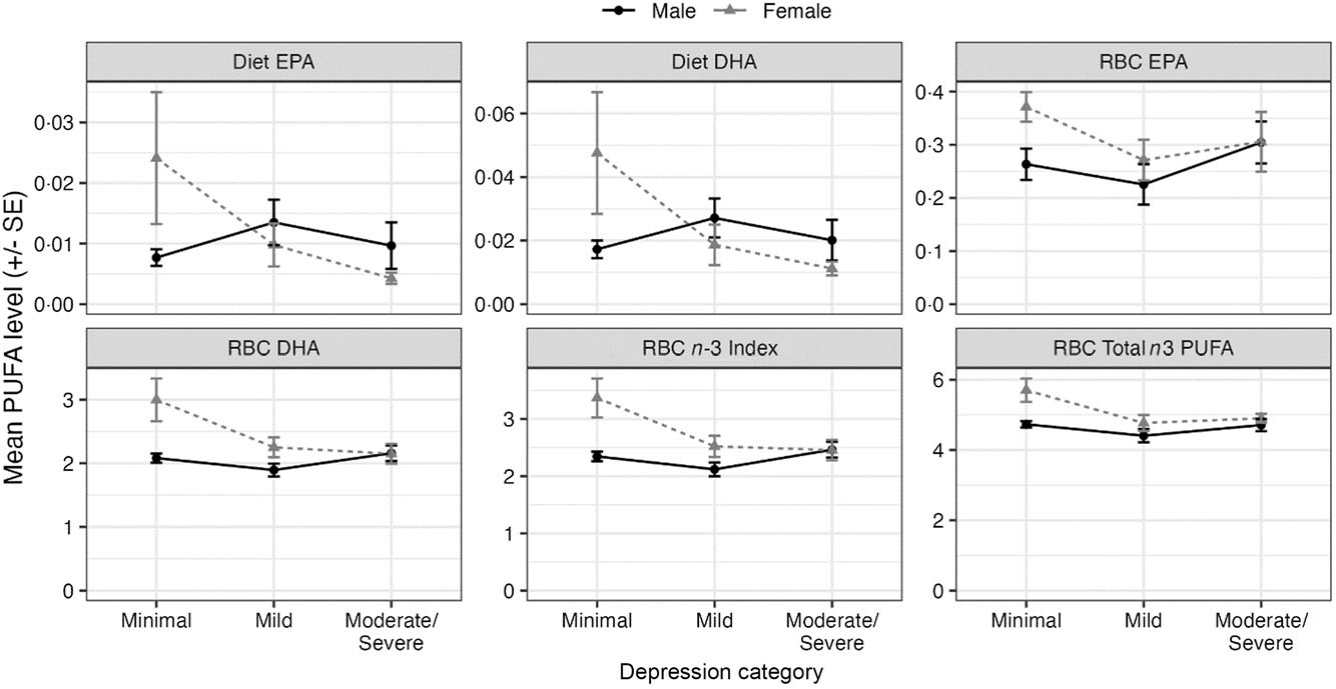
Moderation of relationship of depression with *n*-3 PUFA intake and status by sex. Diet PUFA: % energy; erythrocytes PUFA: % concentration.

About 41 % of participants reported having minimal depression, 32 % had mild depression and 27 % had moderate or severe depression (Table 4). Furthermore, females reported more severe depression symptoms than males (average BDI-II scores 20·4 ± 15·2 *v*. 12·2 ± 10·9; *P* = 0·007). Unadjusted and adjusted analyses did not yield any significant associations between depression category and dietary intakes of *n*-3 PUFA (energy-adjusted) (Table 5). However, significant moderation of this relationship existed by sex for dietary EPA and DHA as well as erythrocytes DHA and *n* 3-index. Specifically, among females, as depression outcomes increased, PUFA intake and status decreased. This significant moderation persisted (Diet EPA: *P* = 0·017, Diet DHA: *P* = 0·008; erythrocytes DHA: *P* = 0·007, *n* 3-index *P* = 0·009), even after adjusting for confounders (age, age first homeless and months without shelter) (Fig. 1 and online Supplementary Table 2). This relationship, however, was not noted among males.

**Table 4.** Depression and anxiety prevalence by gender

	Symptom severity							
	Minimal		Mild		Moderate		Severe	
	*n*	%	*n*	%	*n*	%	*n*	%
Depression								
Total	47	41	36	32	15	13	15	13
Male	40	50	23	29	10	13	7	9
Female	7	21	13	38	5	15	8	24
Anxiety								
Total	62	54	28	25	14	12	9	8
Male	50	63	17	21	10	13	3	4
Female	12	35	11	32	4	12	6	18

*n* 113; Males *n* 80, females *n* 33. Missing BDI/BAI score from one participant.

**Table 5.** Mean dietary and erythrocytes *n*-3 PUFA by depression categories

Fatty acid	Minimal		Mild		Moderate/severe		Unadjusted *P* value	Adjusted† *P* value
	Mean	sd	Mean	sd	Mean	sd		
Diet EPA* (% energy)	0·01	0·01	0·01	0·02	0·01	0·01	0·467	0·481
Diet DHA* (% energy)	0·02	0·03	0·02	0·03	0·02	0·02	0·507	0·519
Erythrocytes EPA (% Area)	0·28	0·18	0·24	0·17	0·31	0·18	0·333	0·320
Erythrocytes DHA (% Area)	2·22	0·62	2·02	0·54	2·15	0·52	0·304	0·274
Erythrocytes n 3-Index (% Area)	2·50*	0·69	2·26	0·63	2·46	0·59	0·250	0·216
Erythrocytes Total *n*-3 PUFA	4·88	0·70	4·54	0·88	4·79	0·62	0·117	0·104

* Energy adjusted.

† Controlling for age, sex, age first homeless and months without shelter.

*P* < 0·05.

*n* varied for Dietary intake and erythrocytes data. Diet: Minimal (*n* 38), Mild (*n* 35), Moderate/Severe (*n* 26).

Erythrocytes: Minimal (*n* 47), Mild (*n* 36), Moderate/Severe (*n* 30).

Erythrocytes n 3-Index = erythrocytes EPA + DHA.

### Anxiety and PUFA

Over half of participants (54 %) reported having minimal anxiety, 25 % had mild anxiety and the remaining 21 % had moderate or severe anxiety (Table 4). Like depressive symptoms, females reported having more severe anxiety symptoms compared with males (average BAI scores 14·0 ± 14·5 *v*. 8·04 ± 9·62; *P* = 0·03). No significant associations were found between erythrocytes status by anxiety category and *n*-3 PUFA intake (energy-adjusted) (Table 6). Similarly, no moderation by sex was found for this relationship (online Supplementary Table 2 and Supplementary Fig. 1).

**Table 6.** Mean dietary and erythrocytes *n*-3 PUFA by anxiety categories

Fatty acid	Minimal		Mild		Moderate/ Severe		Unadjusted *P* value	Adjusted‡ *P* value
	Mean	sd	Mean	sd	Mean	sd		
Diet EPA† (% energy)	0·01	0·01	0·01	0·02	0·01	0·01	0·703	0·710
Diet DHA† (% energy)	0·02	0·03	0·02	0·03	0·02	0·02	0·746	0·751
Erythrocytes EPA (% Area)	0·27	0·18	0·30	0·16	0·27	0·19	0·690	0·681
erythrocytes DHA (% Area)	2·15	0·59	2·08	0·52	2·17	0·6	0·804	0·791
Erythrocytes *n* 3-Index (% Area)	2·42	0·68	2·38	0·57	2·44	0·70	0·938	0·932
Erythrocytes Total *n*-3 PUFA	4·75	0·80	4·80	0·70	4·67	0·71	0·838	0·831

† Energy adjusted.

‡ Controlling for age, sex, age first homeless and months without shelter.

*n* varied for dietary intake and erythrocytes data:

Diet: Minimal (*n* 38), mild (*n* 35), moderate/severe (*n* 26).

Erythrocytes: Minimal (*n* 47), mild (*n* 36), moderate/severe (*n* 30).

Erythrocytes *n* 3-Index = erythrocytes EPA + DHA.

## Discussion

The current study had two aims: to characterise the long-chain PUFA dietary intake and erythrocytes status of homeless youth and to determine whether depression and anxiety relate to n-3 PUFA. As a vulnerable, understudied population, little is known about the dietary intake and erythrocytes status of *n*-3 fatty acids among homeless youth. ALA consumption of study participants generally met the adequate intakes set by the National Academy of Nutrition, with mean ALA intakes of 2·75 g/d for men (adequate intakes: 1·6 g/d) and 1·65 g/d for women (adequate intakes: 1·1 g/d)^(42)^. However, the mean intakes of EPA and DHA for all participants were, respectively, 60 mg/d and 110 mg/d, which is well below the recommended 450–500 mg/d EPA and DHA^(43)^. There were no significant differences in the intake of PUFA by sex except for ARA. Given that homeless youth have limited access and consumption of healthy foods including fish, a primary source of essential fatty acids in the American diet, this inadequate intake is unsurprising. Seafood, particularly fatty fish such as salmon, herring and mackerel, is typically expensive and requires preparation, and fish that is more affordable and accessible for homeless youth is typically white fish that is lower in PUFA^(26)^. Additionally, lower income and younger age have been associated with lower odds of consuming seafood^(44)^. This has negative implications for homeless youth, since essential PUFA cannot be synthesised de novo and must be consumed in the diet.

Given the youths’ low intakes of EPA and DHA, a low biological *n*-3 PUFA status is expected. Mean erythrocytes EPA + DHA composition in this homeless youth cohort (2·42 %) was lower than what has been found in healthy, housed adolescents as well as those with clinical depression: McNamara *et al*. recorded a erythrocytes EPA + DHA composition of 4·24 % for healthy adolescents and a composition of 3·10 % for those with depression^(45)^, while Pottala *et al.* found compositions of 3·71 % and 3·47 %, respectively^(45)^. Both of the aforementioned studies were conducted in urban Midwestern cities similar to the present study’s location. Although dietary intakes of *n*-3 PUFA are correlated with erythrocytes concentrations, additional factors may be contributing to the low biological statuses observed^(47)^. Prolonged psychological stress, depression or anxiety can stimulate dysfunction in the hypothalamic–pituitary–adrenal axis, releasing proinflammatory hormones that promote the oxidation of PUFA^(48,49)^. Since homeless youth live in stressful environments and have high rates of depression and anxiety, these oxidative effects may contribute to the low *n*-3 PUFA status observed in the sample. Additionally, alcohol intake has been found to deplete DHA in the brain^(50)^, and its effects on erythrocytes composition are unknown. Although alcohol intake was not accounted for in this study, its consumption among homeless youth may also contribute to the low biological levels observed.

Compared with housed youth, homeless youth are about twice as likely to suffer from depression^(7)^. Our study shows that one-quarter of homeless youth had moderate or severe depression, and females reported more depressive symptoms than males. These findings validate previous studies of depression prevalence among homeless youth in urban USA cites^(7,8,51)^. One study using the Mini International Neuropsychiatry Interview found that 31·3 % of homeless youth had depression^(51)^, while another study using the Center for Epidemiologic Studies Depression Scale (CES-D) found that 23 % of males and 39 % of females met the cut-off for clinical depression^(7)^. In addition to a high depression prevalence, this also suggests that homeless females may be disproportionally affected by depression. Previous research attempted to explain this observation with the suggestion that females may exhibit greater depressive symptomatology due to increased susceptibility to sexual or physical abuse on the streets compared with males^(4)^, but further research is needed concerning this disparity.

The present study found that dietary intakes and erythrocytes status of *n*-3 PUFA were not associated with depression symptoms. It is unknown whether poor *n*-3 PUFA intake precedes depression or is a result of depression. However, several observational studies have found significant negative associations between fish intake and depression^(27,29,52)^, and mood has been shown to influence diet: individuals with depressive symptomology typically prefer more palatable, high fat, energy-dense foods, which are low in *n*-3 PUFA^(53)^. Biological levels of *n*-3 PUFA have also been previously negatively associated with depression. In a case–control study of adolescents aged 13–18 years, those with depression had significantly lower erythrocytes levels of DHA and ALA but not EPA than controls^(46)^. Similarly, a small study of fourteen control and ten depressed adults by Edwards *et al.* found that EPA, DHA and ALA status were all significant predictors of depression^(52)^. Interestingly, among adolescents who were supplemented with *n*-3s as part of a krill oil trial, depression was not associated with biological levels of DHA, EPA or *n*-3 index^(54)^. The finding that the relationship of depression with *n*-3 PUFA intake and status is moderated by sex, where increased depression was associated with low *n*-3 PUFA among female youth is novel. A similar finding, however, has been noted in elderly Japanese women with overweight or obesity^(55)^.

Although higher rates of anxiety are expected in homeless youth due to trauma, victimisation and unstable living conditions^(2)^, previous studies of homeless youth have reported that most participants experience minimal anxiety symptoms^(56)^. This supports the rates found in the present study: half of participants reported having minimal anxiety symptoms, with females reporting more severe anxious symptoms than males. Poor diets, particularly the Western diet, have been associated with anxiety^(57)^, and homeless youth consume diets that are typically low in fruits, vegetables and fish but high in refined carbohydrates, saturated fat and fast food^(13)^. We observed no relationship between anxiety and *n*-3 PUFA intake and status, which is similar to prior findings. In a cross-sectional, community-based study of women over the age of 20 years, there was no relationship between ALA, EPA or DHA intake and anxiety disorders; however, these women consumed about twice as much *n*-3 PUFA as our participants^(58)^. Similarly, anxiety symptoms were not related to plasma *n*-3 PUFA concentrations in a sample of middle-aged, housed adults^(59)^.

The study strengths include the use of an objective blood biomarker of long-term fatty acid status in our assessment in addition to a validated self-reported measure of dietary intake to assess *n*-3 consumption. However, we acknowledge that there are limitations as well. A key limitation of this study, as with most research among homeless youth, is the cross-sectional nature of the data. Homeless youth are a transient, difficult-to-reach population, making longitudinal studies impractical. Therefore, the cross-sectional analysis can only suggest an association of depression and anxiety with *n*-3 PUFA intake and status; it fails to show causality. The difficult-to-reach characteristic of homeless youth also necessitated the use of an FFQ with this population, instead of using diet record or multiple administrations of 24 h dietary recalls, to determine usual dietary intake. FFQ are prone to random and systematic errors; however, they have been shown to be more valid for estimating long-chain PUFA in various populations^(60–62)^. FFQ-derived dietary PUFA are not always correlated with blood levels^(63)^. However, the inclusion of PUFA blood biomarker assessment, as done in the current study, provides an unbiased long-term measure of PUFA, devoid of recall bias. Second, the study sample is a convenience sample of homeless youth who utilise drop-in centres and therefore may not be representative of all youth experiencing homelessness in this midwestern city. Additionally, due to the small sample size, our findings may not be generalisable. Nonetheless, the findings in this study are significant as they add to the literature on the nutritional vulnerabilities of a transient and difficult to reach population. Larger studies are warranted to confirm these findings. As a vulnerable population, homeless youth disproportionately experience metal health challenges and lack adequate intake of foods including fruits, vegetable, whole grains and healthy fats^(11,13,14)^. Current evidence continues to emphasise the importance of a healthy diet and nutrition in preventing metal health conditions and/or ameliorating the effects of these conditions^(64–67)^. The current study further highlights the mental health and dietary needs of homeless young adults, suggesting the need for increased focus on essential fatty acids intake among homeless youth, especially females.

## Supplementary material

For supplementary material accompanying this paper visit https://doi.org/10.1017/S000711452300212X. click here to view supplementary material
