# Supplementary material for: Mental health in society’s margins: poor n-3 PUFA intake and psychological well-being of homeless youth
Source: Br J Nutr. 2023 Sep 22;131(4):698–706. doi: 10.1017/S000711452300212X (PMC10803817; doi:10.1017/S000711452300212X)
Supplement: Supplementary file 1 [file S000711452300212Xsup001.docx]

Supplemental Table 1: Association between PUFA and Depression by Sex Interaction

|  | **BDI *Sex Interaction** | |
| --- | --- | --- |
| **Fatty Acid** | **Unadjusted p-value** | **Adjusted p-value^b^** |
| Diet EPA^a^ | 0.016 | 0.017 |
| Diet DHA^a^ | 0.008 | 0.008 |
| RBC EPA | 0.541 | 0.562 |
| RBC DHA | 0.008 | 0.007 |
| RBC EPA + DHA | 0.010 | 0.009 |
| RBC Totaln3PUFA | 0.124 | 0.088 |

^a^ Energy adjusted

^b^ Controlling for age, age first homeless, and months without shelter

Supplemental Table 2: Association between PUFA and Anxiety by Sex Interaction

|  | **BAI *Sex Interaction** | |
| --- | --- | --- |
| **Fatty Acid** | **Unadjusted p-value** | **Adjusted p-value^b^** |
| Diet EPA^a^ | 0.401 | 0.651 |
| Diet DHA^a^ | 0.324 | 0.582 |
| RBC EPA | 0.558 | 0.714 |
| RBC DHA | 0.203 | 0.403 |
| RBC EPA + DHA | 0.196 | 0.447 |
| RBC Totaln3PUFA | 0.778 | 0.906 |

^a^ Energy adjusted

^b^ Controlling for age, age first homeless, and months without shelter


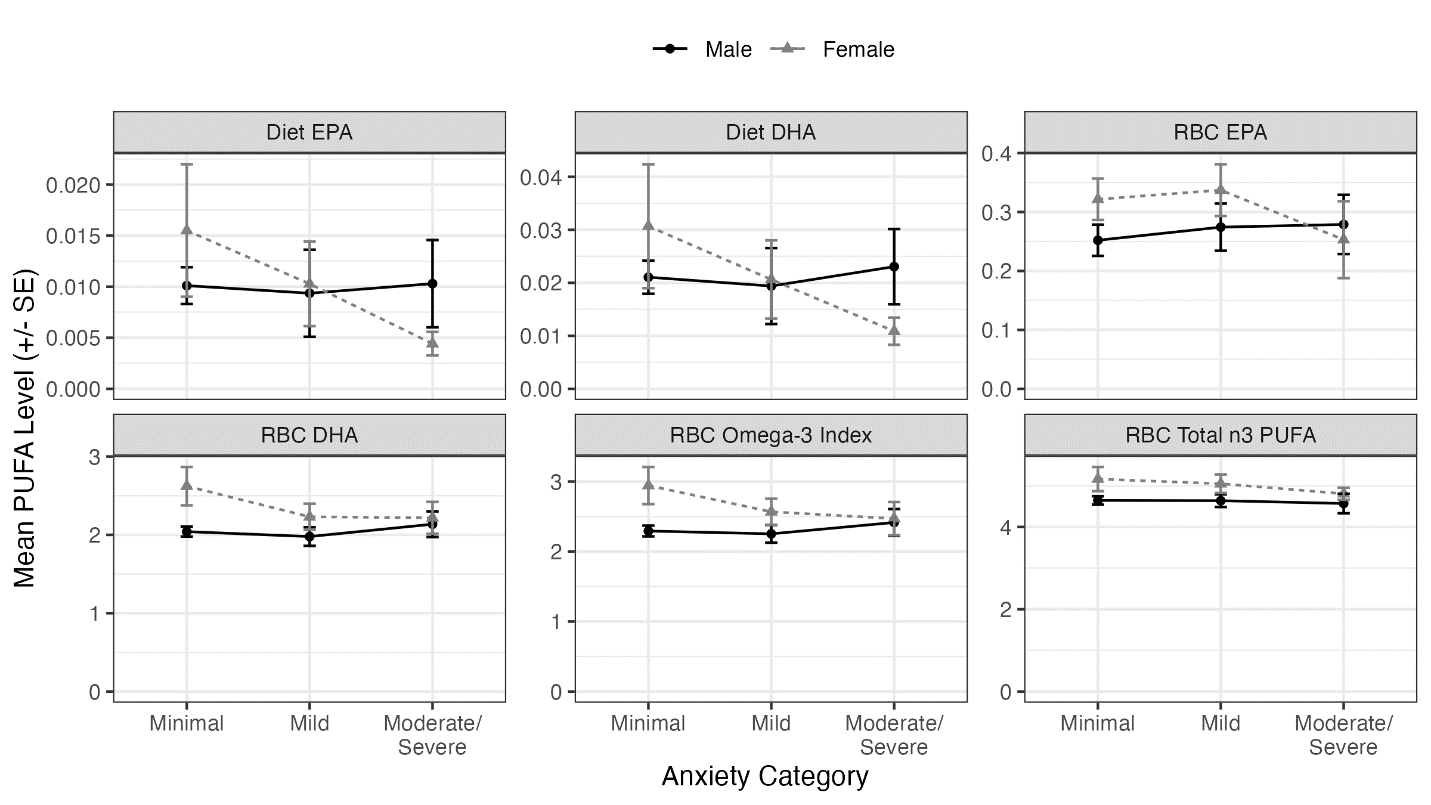


Supplemental Figure 1: Moderation of relationship of anxiety with n-3 PUFA intake and status by sex.

Diet PUFAs: % energy; RBC PUFA:% concentration.
